# Electrospun Nanofibers With pH-Responsive Coatings for Control of Release Kinetics

**DOI:** 10.3389/fbioe.2019.00309

**Published:** 2019-11-27

**Authors:** Sezin Sayin, Ali Tufani, Melis Emanet, Giada Graziana Genchi, Ozlem Sen, Sepideh Shemshad, Ece Ozdemir, Gianni Ciofani, Gozde Ozaydin Ince

**Affiliations:** ^1^Materials Science and Nano Engineering Department, Faculty of Engineering and Natural Sciences, Sabanci University, Istanbul, Turkey; ^2^Istituto Italiano di Tecnologia, Smart Bio-Interfaces, Pontedera, Italy; ^3^Department of Mechanical and Aerospace Engineering, Politecnico di Torino, Turin, Italy; ^4^Sabanci University Nanotechnology Research and Application Center (SUNUM), Sabanci University, Istanbul, Turkey; ^5^Center of Excellence for Functional Surfaces and Interfaces (EFSUN), Sabanci University, Istanbul, Turkey

**Keywords:** initiated chemical vapor deposition, electrospinning, controlled drug delivery, pH responsive polymers, polymer-coated nanofibers

## Abstract

Functional and stimuli-responsive nanofibers with an enhanced surface area/volume ratio provide controlled and triggered drug release with higher efficacy. In this study, chemotherapeutic agent Rose Bengal (RB) (4,5,6,7-tetrachloro-2′, 4′,5′,7′-tetraiodofluoresceindisodium)-loaded water-soluble polyvinyl alcohol (PVA) nanofibers were synthesized by using the electrospinning method. A thin layer of poly(4-vinylpyridine-*co*-ethylene glycol dimethacrylate) p(4VP-*co*-EGDMA) was deposited on the RB-loaded nanofibers (PVA-RB) via initiated chemical vapor deposition (iCVD), coating the fiber surfaces to provide controllable solubility and pH response to the nanofibers. The uncoated and [p(4VP-co-EGDMA)-PVA] coated PVA-RB nanofiber mats were studied at different pH values to analyze their degradation and drug release profiles. The coated nanofibers demonstrated high stability at neutral and basic pH values for long incubation durations of 72 h, whereas the uncoated nanofibers dissolved in <2 h. The drug release studies showed that the RB release from coated PVA-RB nanofibers was higher at neutral and basic pH values, and proportional to the pH of the solution, whereas the degradation and RB release rates from the uncoated PVA-RB nanofibers were significantly higher and did not depend on the pH of environment. Further analysis of the release kinetics using the Peppas model showed that while polymer swelling and dissolution were the dominant mechanisms for the uncoated nanofibers, for the coated nanofibers, Fickian diffusion was the dominant release mechanism. The biocompatibility and therapeutic efficiency of the coated PVA-RB nanofibers against brain cancer was investigated on glioblastoma multiforme cancer cells (U87MG). The coated PVA nanofibers were observed to be highly biocompatible, and they significantly stimulated the ROS production in cells, increasing apoptosis. These promising results confirmed the therapeutic activity of the coated PVA-RB nanofibers on brain cancer cells, and encouraged their further evaluation as drug carrier structures in brain cancer treatment.

## Introduction

Implantable patches for drug delivery have gained attention in recent years. In particular, these patches are used in site-specific, targeted delivery with enhanced sustained release of drug molecules from biodegradable materials (Brown and Crawford, 2002; LaPorte et al., 2005). These devices have been developed to overcome the common challenges in drug delivery, such as achieving systematic delivery with therapeutically effective drug concentrations at the specified target. Theeuwes and Nelson (2004) developed a bilayered, patch-based device, which provided drug delivery directly to the organ surface. The biocompatible, drug-impermeable first layer acted as the drug reservoir, while the drug-permeable second layer allowed drug delivery directly to the organ. Nelson et al. (2003), on the other hand, introduced a biodegradable fiber implant for drug release, which involved three-dimensional matrices of predefined non-homogeneous patterned polymeric fibers.

Electrospun nanofibers as polymeric nanocarriers are widely used in drug delivery systems (DDS) because they have high loading and encapsulation efficiency and they can be easily produced in a cost-effective manner (Chakraborty et al., 2009; Wang et al., 2010). Blending a polymer solution with a therapeutic agent before electrospinning is the most common technique for encapsulation. The distribution of drug molecules and morphology of fibers are the main factors affecting the release behavior (Kenawy et al., 2002; Zamani et al., 2013; Tipduangta et al., 2015).

As chemotherapeutic DDS, electrospun nanofibers are promising due to high drug loading capacities. Poly(ethylene glycol)–poly(L-lactic acid) (PEG–PLLA) diblock copolymer fibers loaded with an antineoplastic drug BCNU (1,3-bis(2-chloroethyl)-1-nitrosourea) were fabricated to obtain controllable drug delivery directly to the tumor microenvironment (Xu et al., 2006). High antitumor activity for longer periods of time (72 h) was observed when the BCNU release was from the PEG–PLLA fibers whereas with pristine BCNU, loss of cytotoxic activity was observed in the same period of time due to the short half-life of the drug (Xu et al., 2006). Sharma et al. (2013) loaded insulin to poly(vinyl alcohol) PVA and sodium alginate nanofiber-based patch for anti-diabetic drug delivery. *In vivo* test of the patch on male Wistar rats showed that the drug molecules were released in their pharmacologically active states without any deterioration. In another study, for the treatment of glaucoma disease, Garg et al. (2014) used PVA and polycaprolactone (PCL) fiber mats loaded with timolol maleate and dorzolamide hydrochloride as model drugs, achieving a very high drug entrapment efficiency of ~100%.

Although electrospun fibers are very promising with respect to drug delivery, in post-operation cancer treatment applications, controlling the initial burst release and tuning the release kinetics from fibers are the main challenges yet to be overcome (Thakkar and Misra, 2017). In order to defeat these challenges during cancer treatments, coaxial nanofibers with core-shell structures have been introduced due to their effectiveness in drug incorporation into nanofibers as reservoir-type drug delivery carriers (He et al., 2006). Recent studies include concentric spinneret electrospinning method for coaxial nanofiber formation. These core-shell nanocarriers are used mostly to control the sustained drug delivery (He et al., 2006; Zupancic et al., 2016), to release both hydrophilic and hydrophobic drugs from the same system (Oliveira et al., 2015), to enhance implant osseointegration and to prevent implant infections (Song et al., 2013), and to obtain bi-component, surface-modified, and functional graded nanofibers (Zhang et al., 2004). Another method that has been used to overcome these shortcomings is direct deposition onto nanofibers for the production of coaxial structures (Chunder et al., 2007). Layer-by-layer (LBL) deposition (Sakai et al., 2009; Li et al., 2012; Croisier et al., 2014) and vapor phase methods, such as chemical vapor deposition (CVD) (Zeng et al., 2005) are some examples.

Although studies reported have included controlled drug release from various polymeric nanofibers, stimuli-responsive and cross-linked coatings on these nanofibers have not been quite examined. In this paper, fabrication of polymeric mat with an outer coating layer for sustained release of Rose Bengal (RB) as a chemotherapeutic drug was reported. PVA polymer with RB solution was electrospun to form blend fibers. PVA is used as the polymer matrix due to its biocompatibility and biodegradable nature, leading to its wide utilization in drug delivery applications (Huang and Rhim, 1993; Taepaiboon et al., 2006; Kenawy et al., 2007; Yang et al., 2007; Jannesari et al., 2011; Bazhban et al., 2013; Li et al., 2013; Jalvandi et al., 2017). Meanwhile, RB (4,5,6,7-tetrachloro-2′,4′,5′,7′-tetraiodofluoresceindisodium) is used as the chemotherapeutic drug which is a water-soluble, photosensitive, synthetic dye used for diagnostics exhibiting cytotoxicity in various cancer types, such as brain cancer (Tserkovsky et al., 2012), colorectal cancer cells (Qin et al., 2017), melanoma and breast cancer cells (Toomey et al., 2013), and ovarian and adenovirus-transformed embryonic kidney cancer cells (Koevary, 2012). In order to provide additional functionalities to the electrospun fibers, surface of the fiber mats was coated with poly(4-vinylpyridine-*co*-ethylene glycol dimethacrylate) [p(4VP-co-EGDMA)], a pH-sensitive polymer, via initiated chemical vapor deposition (iCVD). iCVD is an all-dry, free-radical polymerization method that allows polymerization directly on the substrate surface, initiated by thermally decomposed radicals reacting with the monomer molecules adsorbed on the substrate (Lau and Gleason, 2006; Ozaydin-Ince et al., 2011). It is advantageous because of its conformal coating ability on high-aspect-ratio surfaces, which preserves the film thickness throughout the surface topography (Ozaydin-Ince et al., 2011; Armagan and Ince, 2015).

In the study reported here, RB release from the p(4VP-*co*-EGDMA)-coated PVA-RB nanofibers was investigated at different pH values, and the effect of the pH-responsive polymer coating on the release performance was studied. The degradation profiles of the nanofibers at these pH values were investigated to reveal their stability in cellular environment for their potential drug carrier activity. Moreover, the time-dependent anti-cancer activity of the coated PVA-RB nanofibers was studied on glioblastoma multiforme brain cancer cells (U87MG). The effects on cell viability were investigated as a preliminary study before exploring the therapeutic effect of the fibers via DNA quantification by using Pico Green dye. The intracellular RB localization and conformation of the cytoskeleton structure of cells was monitored by using confocal microscopy. The intracellular stress and death mechanism of the cells were investigated to analyze their cellular response against RB exposure. The significantly decreased cell viability and increased intracellular ROS production confirmed the increased apoptosis.

## Materials and Methods

### Materials

PVA (MW 85,000–124,000, 87–89% hydrolyzed, Aldrich) and Tserkovsky Bengal sodium salt (dye content ~90%, Aldrich) were used in electrospinning for fiber synthesis. The monomer 4VP (95%, Aldrich), the cross-linker EGDMA (98%, Aldrich), and the initiator tertbutyl peroxide (TBPO, 98%, Aldrich) were used without purification. Phosphate-buffered saline (PBS, Aldrich) was utilized for the release studies.

### Preparation of PVA-RB Nanofiber Mats

Ten wt% PVA solution was obtained by dissolving PVA in distilled water at 70°C and stirred continuously for 4 h. Homogeneous solution was observed after stirring overnight at room temperature, at 500 rpm. PVA-RB solution was prepared by blending 100 mg/ml RB distilled water and 10 wt% PVA solutions in RB:PVA 1:5 ratio. The loading capacity of the nanofibers was calculated to be ~16.7%.

Electrospinning setup included syringe pump, stainless steel spinneret needle, high voltage supply, 10 × 10 cm collector, and 2 ml syringe. The distance between the needle and collector was kept at 15 cm, and the voltage was applied at 8 kV. Flow rates for both PVA and PVA-RB blend solutions were adjusted as 0.3 ml/h.

### P(4VP-*co*-EGDMA) Coating of PVA-RB Nanofibers

**Figure 1:**
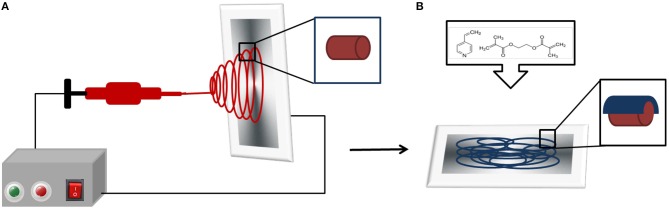
Schematic representation of the synthesis of the coated and uncoated nanofiber mats. **(A)** Electrospinning method used for the fabrication of the RB-loaded nanofiber mats. **(B)** iCVD p(4VP*-co*-EGDMA) deposition on the electrospun RB-loaded nanofiber mats. The inset shows the polymer-coated nanofibers.

P(4VP-*co*-EGDMA) was conformally deposited on both sides of nanofiber mats at a thickness of ~70 nm by using iCVD. The cross-linker EGDMA was heated to 95°C in a metal jar, and 4VP was kept at room temperature. The initiator was delivered to the system at a flow rate of 1 sccm while the flow rate of N_2_ gas was 1.1 sccm. The filament and substrate temperatures were at 240 and 25°C, respectively, throughout the deposition. Reaction pressure was fixed at 600 mTorr. Flow rates of 4VP and EGDMA were 2.73 and 0.14 sccm, respectively. Si wafers were coated simultaneously as the fiber mats, to be used in ellipsometric swelling experiments. Figure 1 shows the schematic of the coated and uncoated nanofiber mat fabrication process.

### Characterization of PVA, PVA-RB, Coated PVA, and Coated PVA-RB Nanofibers

The PVA, PVA-RB, p(4VP-co-EGDMA)-coated PVA, and p(4VP-co-EGDMA)-coated PVA-RB nanofibers were imaged by field emission scanning electron microscopy (Zeiss, Leo Supra VP35) with an accelerating voltage of 4 kV. Chemical characterization of the fibers was performed by Fourier transform infrared spectroscopy (Thermo-Fisher Scientific, Nicolet iS10 FTIR) with 62 scans and the resolution of 4 cm^−1^ over the range of 800–4,000 cm^−1^.

XPS analysis was done by using Thermo Scientific K-Alpha spectrometer by an aluminum anode (Al Kα = 1,468.3 eV) at an electron take-off angle of 90° (between the sample surface and the axis of the analyzer lens). The spectra were recorded using an Avantage 5.9 data system. The binding energy scale was calibrated by assigning the C1s signal at 284.5 eV.

Swelling behavior of the iCVD polymer film on Si wafer was investigated via a spectroscopic ellipsometer (M2000D J.A. Woollam Co. Inc.) in a liquid cell stage at room temperature at pH values of 4, 6.5, and 9. Dynamic thickness measurements were performed at 75° nominal angle of incidence within the wavelength range of 315–718 nm for 30 min. Swelling percentages were calculated by using the formula (*t* – *t*_0_)^*^100/*t*_0_, where *t* is the thickness of the swollen and *t*_0_ is the thickness of the dry samples.

### Degradation of PVA, PVA-RB, Coated PVA, and Coated PVA-RB Nanofibers

The degradation behavior of PVA, PVA-RB, coated PVA, and coated PVA-RB nanofibers was tested in PBS solution at several pH values (pH 4.5, 6.5, and 9) following 24 and 72 h incubation times. The morphology of the fibers was visualized by monitoring the samples using SEM (Helios Nano-Lab 600i FIB/SEM, FEI). Samples were incubated in 2-ml PBS solutions adjusted at pH 4, 6.5, and 9 for 24 and 72 h by shaking at room temperature. Following the incubations, the samples were dried under vacuum conditions for 24 h and prepared for SEM imaging. SEM imaging was carried out on samples gold-sputtered for 25 s at 60 nA, obtaining a 3 nm-thick conductive layer over the nanofibers.

### Release From PVA, PVA-RB, Coated PVA, and Coated PVA-RB Nanofibers

Coated and uncoated electrospun nanofibers were cut into 1 × 2 cm pieces and put in 10 ml PBS solutions separately at pH 4, 6.5, and 9 and placed on a shaker for the release experiments. UV-Vis measurements were performed using NanoDrop UV-Vis spectrophotometer (Thermo Scientific NanoDrop 2000c). For each measurement, 100 μl of PBS solution was removed from the solution and replaced with fresh PBS solution in the release medium. For RB detection, the UV absorbance peak at 550 nm was used. For each release experiment, eight sets of new samples were prepared and UV-Vis measurements were repeated at least twice on each sample.

The amount of RB loaded in the nanofibers was determined by dissolving the uncoated fibers completely and measuring the RB concentration of the solution. Release percentages of the coated nanofibers were obtained by normalizing the concentration of RB released from the nanofibers by the amount loaded.

### Cellular Experiments of Coated PVA and Coated PVA-RB Nanofibers

#### Cell Culture

U87-MG tumor cells (from ATCC) were cultured under high glucose Dulbecco's Modified Eagle's Medium, added with 10% fetal bovine serum, 2 mM L-glutamine, and 100 U/ml penicillin−100 μg/ml streptomycin (all reagents were from Sigma-Aldrich) in saturated humidity, 5% CO_2_ atmosphere. Experiments were performed by seeding 30,000 U87MG cells/cm^2^ in 24-well multiwell polystyrene (PS) plates and by incubating the cultures with 0.8 × 0.8 cm^2^ coated PVA and coated PVA-RB nanofiber samples. For immunocytochemistry, cells were seeded on 0.8 × 0.8 cm^2^ Ibidi films made hydrophilic by O_2_ plasma (50 W, 25 sccm for 60 s) in a Colibrì reactor (Gambetti). Cells were exposed to nanofiber samples after overnight incubation.

#### Proliferation of Coated PVA and Coated PVA-RB Nanofiber-Exposed Cells

Cell proliferation was investigated by Pico Green assay (from Invitrogen) at 24 and 72 h from incubation with coated PVA and coated PVA-RB nanofiber samples. The assay enables ds-DNA quantification in solution by fluorescence measurement and requires cell lysis in a fixed volume of water (500 μl) by three cycles of freezing and thawing. To the purpose, 100 μl of working solution was mixed to 50 μl of cell lysate and to 150 μl of solution containing the PicoGreen dye. Samples were incubated in the dark for 10 min, and finally fluorescence was read with a microplate reader (Perkin Elmer Victor X3, λ_ex_ = 485 nm; λ_em_ = 535 nm).

#### Immunocytochemistry of Coated PVA and Coated PVA-RB Nanofiber-Exposed Cells

U87MG cells were washed with PBS with calcium and magnesium, and fixed with 4% paraformaldehyde (from Sigma-Aldrich) in PBS for 20 min at 4°C. Then, cells were again rinsed with PBS and permeabilized with 0.1% Triton X-100 (from Sigma-Aldrich) in PBS for 30 min. Specific binding sites were saturated with 10% goat serum (GS, from Thermo Scientific) in PBS for 1 h at 37°C. Then, cells were incubated with a 10% GS solution containing a rabbit polyclonal IgG primary antibody against Ki-67 (from Millipore, 1:100 diluted) for 3 h at 37°C. Samples were then rinsed four times with 10% GS (5 min each rinse), and cells were incubated with a 10% GS solution containing a goat polyclonal IgG secondary antibody (from Thermo Scientific, 1:200 diluted) and 1 μM DAPI for 45 min at 37°C. After one rinse with 0.45 M NaCl PBS and with PBS, samples underwent fluorescence imaging. The number of Ki-67 immunopositive nuclei (*n*) and the total number of nuclei (*m*) were semi-automatically counted with ImageJ software and the *n*/*m* ratio (expressed as average ± standard deviation) was calculated and plotted. For statistical reasons, 10 images at low magnification were analyzed.

#### Reactive Oxygen Species (ROS) and Cell Death Mechanism Detection of Coated PVA and Coated PVA-RB Nanofiber-Exposed Cells

To quantitatively assess oxidative stress, cell cultures were stained with CellROX Green reagent (from Invitrogen) at 24 and 72 h from incubation with coated PVA and coated PVA-RB nanofiber samples. To the purpose, cells were rinsed with PBS without calcium and magnesium, and suspended by 1 min incubation with 0.05% trypsin (from Sigma-Aldrich). Cell pellets were obtained with 7 min centrifugation at 1,000 *g*, and staining was performed for 15 min in the dark on 200 μl of cell suspension in PBS with calcium and magnesium, containing 5 μM CellRox Green reagent. To quantitatively study cell viability, cell cultures were stained with FITC Annexin V/Dead Cell Apoptosis kit (from Invitrogen) by incubating 100 μl of cell suspension in 1× annexin-binding buffer, added with 5 μl of FITC Annexin V solution and with 1 μl of 100 μg/ml of propidium iodide. Staining was performed for 15 min in the dark. Flow cytometry was conducted with CytoFLEX platform (Beckman Coulter, λ_ex_ = 488 nm; 500 nm < λ_em_ < 560 nm).

#### Statistical Analysis

Each experiment was carried out at least three times. Statistical analysis was carried out using an unpaired Student's *t*-test. The data are expressed as the mean ± standard deviation (SD). Comparison with a *p*-value below 0.01 and 0.05 was considered statistically significant.

## Results and Discussion

### Characterization of PVA, PVA-RB, Coated PVA, and Coated PVA-RB Nanofibers

**Figure 2:**
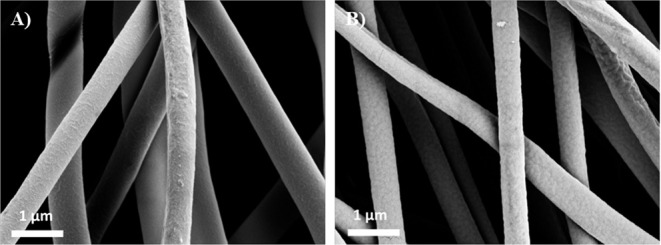
SEM images of **(A)** uncoated PVA-RB nanofibers and **(B)** p(4VP-*co*-EGDMA)-coated PVA-RB nanofibers.

SEM images of (a) uncoated PVA-RB nanofibers and (b) coated PVA-RB nanofibers are shown in Figure 2. The average diameter of pure PVA nanofibers is ~425 ± 22 nm, whereas RB-blended PVA fibers are 523 ± 39 nm in diameter. Increased concentration in solution with RB addition resulted in larger diameters observed (Aljehani et al., 2014). The iCVD polymer coating on the fibers increased the average diameter to 587 ± 63 nm, confirming that the thickness of the polymer coating is ~65 ± 5 nm.

As confirmed by the SEM images, the structures of the nanofibers were not damaged after the iCVD process and the fibers could be conformally coated with the polymer layer, enabling the fabrication of p(4VP-*co*-EGDMA) coated PVA-RB nanofibers.

Swelling percentage of p(4VP-*co*-EGDMA) thin films on Si wafers was determined to be 62 ± 10% at pH 4, whereas at both pH 6.5 and pH 9, swelling was <5%, confirming the pH response of the polymer coating. The mesh sizes of the polymer coatings at pH values of 4, 6.5, and 9 were calculated as 1.16, 0.69, and 0.69 nm, respectively (Supporting Information). Swelling of the polymer at low pH values is attributed to the protonation of 4VP in the acidic environment, causing the chains to stretch due to electrostatic repulsion. Deprotonation of the polymer chains at high pH values, on the other hand, leads to the collapsed state observed (Li et al., 2008, Wang et al., 2013).

**Figure 3:**
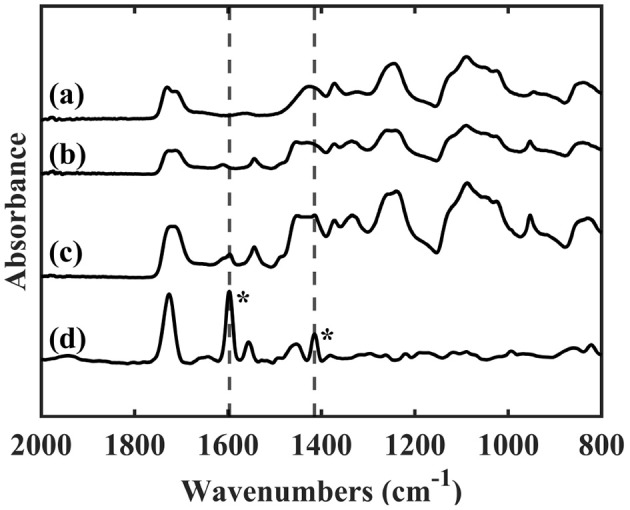
FTIR spectra of **(a)** PVA nanofibers, **(b)** PVA-RB nanofibers, **(c)** p(4VP-*co*-EGDMA)-coated PVA-RB nanofibers, and **(d)** p(4VP-*co*-EGDMA) thin film on Si wafer. The bands at 1,597 and 1,415 cm^−1^ corresponding to the pyridine ring vibrations (indicated with asterisk) can also be observed on the coated PVA-RB nanofibers **(c)** confirming the presence of the polymer coating.

FTIR spectra of pure PVA, PVA-RB, and p(4VP-*co*-EGDMA)-coated PVA-RB nanofibers are shown in Figure 3. The absorption bands at 1,734, 1,429, and 1,091 cm^-1^ are related to the C = O double bond, CH_2_, and C–O–C stretching modes of pure PVA nanofibers, respectively (Figure 3a) (Mansur et al., 2008). The bands at 1,717, 1,457, and 1,091 cm^–1^ belong to the C = O double bond, CH_2_, and C–O–C stretching modes of PVA for RB-PVA nanofibers. Additional bands in RB-PVA blend nanofibers at 1,615 cm^–1^ belongs to the C = O double bond of carbonyl group whereas those at 1,547, 1,444, and 1,337 cm^-1^ correspond to C = C double bonds of aromatic rings (Figure 3b) (Dabrzalska et al., 2016). The absorption band in coated PVA-RB nanofibers at 1,719 cm^-1^ indicates CO double bond stretching of EGDMA, whereas 1,599, 1,547, and 1,417 cm^-1^ belong to pyridine ring vibration and 1,060 cm^-1^ of 4VP and 954 cm^–1^ indicate in-plane and out-of-plane CH bending of 4VP (Figure 3c) (Bayari and Yurdakul, 2000; Lau and Gleason, 2007), confirming the presence of the polymer coatings on the nanofibers.

**Figure 4:**
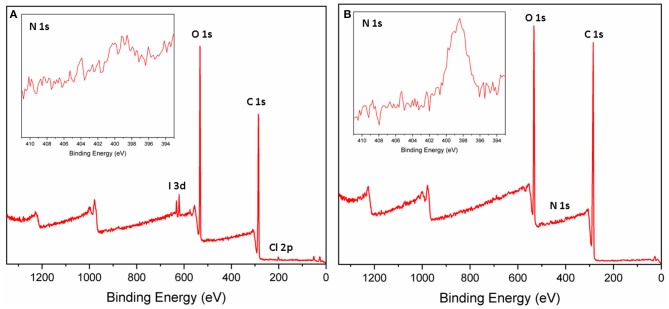
XPS spectra of the **(A)** uncoated and **(B)** coated PVA-RB nanofibers. N1s spectrum (inset figures) observed in **(B)** confirms the presence of the coating.

X-ray photoelectron spectroscopy (XPS) was used to determine the surface composition of the PVA-RB nanofibers within 5–10 nm from the surface. Figures 4A,B show XPS survey scans and N1s spectrum (inset figures) of PVA-RB nanofibers without and with iCVD coating, respectively. Peaks in Figure 4A at 620, 532, 285, and 199 eV correspond to I3d, O1s, C1s, and Cl2p, respectively, and indicate elemental composition of PVA-RB. From the spectrum, the atomic ratios of the elements on the surface of the PVA-RB nanofibers are estimated to be 67.64% C, 31.52% O, 0.35% I, and 0.49% Cl.

According to the XPS spectrum of the polymer-coated nanofibers, depicted in Figure 4B, peaks at 530, 399, and 285 eV correspond to O1s, N1s, and O1s, respectively. The atomic ratios of elements on the surface of the coated nanofibers are calculated to be 73.83% C, 25.37% O, and 0.79% N. The I and Cl peaks could not be detected, possibly due to the presence of the coating. The presence of N1s peak observed only in the coated nanofibers indicates the presence of N-containing p(4VP-*co*-EGDMA) coating on the nanofiber surfaces. The low percentages can be attributed to the fact that the coatings cover mostly the nanofibers, which are closer to the surface of the mat, leaving the nanofibers below the surface uncoated. Therefore, XPS analysis confirms the presence of a polymer coating on the nanofibers exposed to the surface.

### Degradation of PVA, PVA-RB, Coated PVA, and Coated PVA-RB Nanofibers

**Figure 5:**
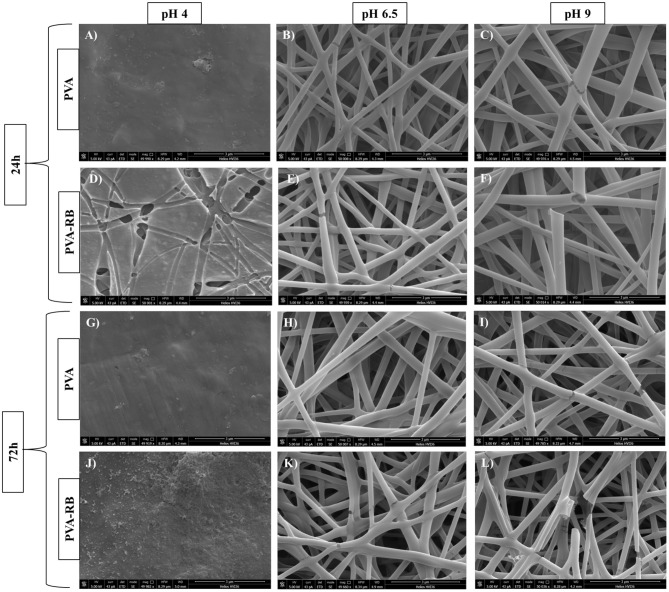
SEM images of coated PVA **(A–C)** and coated PVA-RB **(D–F)** nanofibers following 24 h of incubation, and coated PVA **(G–I)** and coated PVA-RB **(J–L)** nanofibers following 72 h of incubation in solutions at pH 4, 6.5, and 9, respectively.

The degradation behaviors of coated PVA and coated PVA-RB nanofibers were investigated at several pH values (4, 6.5, and 9) of PBS at increasing incubation times, as shown by SEM images (Figure 5). Uncoated PVA-RB and PVA nanofibers completely dissolved in <2 h at all pH values tested. Therefore, long-term stability tests were performed on the coated PVA and coated PVA-RB nanofibers. The SEM images show that the morphology of the coated PVA-RB and coated PVA nanofibers was similarly affected following the incubation.

The coated nanofibers were not stable at low pH values at incubation times longer than 12 h (Figures 5A,D,G,J). On the other hand, the coated nanofibers showed significantly high stability in PBS solutions at pH 6.5 (Figures 5B,E,H,K) and at pH 9 (Figures 5C,F,I,L). The pH-dependent degradation could be explained by the protonation of pyridine groups of p(4VP) at low pH values (pH 4) that lead to swelling of the p(4VP-*co*-EGDMA) coatings on the PVA nanofibers (Li et al., 2008). The swelling of the polymer coating enhanced the diffusion of the acidic solution through coating, resulting in the complete degradation of PVA nanofibers. Higher stability of the coated PVA nanofibers observed at high pH values (pH 6.5 and 9) could be attributed to the collapsed state of the polymer chains, which reduces the diffusion of the solution medium through the coating, hindering degradation of the PVA nanofibers.

### Drug Release From p(4P-*co*-EGDMA)-Coated PVA-RB Nanofibers

**Figure 6:**
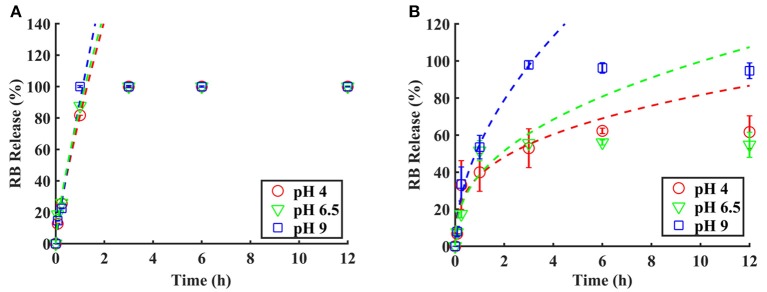
RB release from **(A)** uncoated PVA-RB fibers and **(B)** coated PVA-RB fibers in PBS solutions at pH 4, 6.5, and 9. Data fitting was performed up to 60% of RB release using Peppas equation.

RB release from the polymer-coated and uncoated PVA-RB nanofibers was investigated in PBS solution at pH 4, 6.5, and 9 (Figure 6). Release experiments from the PVA-RB nanofibers showed that more than 80% of the RB was released in 1 h with no significant dependence of the release rate on the pH of the medium (Figure 6A).

The kinetics of the RB release from the coated PVA-RB nanofibers, on the other hand, was strongly affected by the pH of the medium (Figure 6B). At all pH values, the overall release was <60% at the end of 1 h, indicating slower early time release kinetics due to the coating layer on the fibers. As the pH decreased, slower release kinetics was observed and the overall release percentages at the end of the experiments were lower at low pH. While 98% of the RB was released at the end of 6 h at pH 9, only 55% was released in 6 h at pH 4. The smaller mesh sizes of the polymer coatings at low and high pH values, compared to the size of RB molecules (hydrodynamic radius of ~1.28 nm), indicate that the release from the uncoated or partially dissolved nanofibers dominates the release and that the release through the polymer coatings is negligible.

The fast release rates and higher release percentages of RB obtained at pH 9 compared to the release rates at lower pH values can be attributed to smaller diameters of the nanofibers due to the collapsed state of the polymer coating, which leads to the increased free volume in the electrospun mat, resulting in improved release rates. At lower pH conditions, on the other hand, the swollen polymer coating of the nanofibers reduces the free volume in the mat and entraps the RB molecules, decreasing the overall release percentages and release rates. The prolonged release observed is, thus, caused by the longer paths the RB molecules have to diffuse through due to swollen polymer.

In addition to the changes in the free volume, the electrostatic interactions between the polymer coating and the released RB molecules may also contribute to the pH dependence of the release profiles at early times. Protonation of pyridine groups of p(4VP), which has a pKa in the range 4.5–4.7, occurs at lower pH values, leading to an electrostatic interaction between the protonated pyridine groups and the RB molecules. This attractive interaction also contributes to the reduced release percentages observed at pH 4.

A transient behavior is observed at pH 6.5 with a faster early time release kinetics due to the collapsed polymer coating on the fibers, resulting in larger free space and shorter diffusion lengths for the dye molecules. However, overall release percentages at the end of 12 h are comparable to the release percentages at pH 4, but lower than the values obtained at pH 9.

The early time release kinetics of the polymer-coated and uncoated PVA-RB fibers were investigated using the semi-empirical Peppas model, which includes relaxation and phase changes of the polymer matrix in addition to the diffusion of the drug molecules. The Peppas model in its simplest form is given by Korsmeyer et al. (1983):

(1) $$\begin{array}{l} \frac{M_{t}}{M_{\infty }}=at^{n} \end{array}$$

where *M*_*t*_ is the amount of drug released at time *t, M*_∞_ is total amount of drug loaded, “*a*” is a constant that depends on the structure and geometry of the of drug-polymer system, and “*n*” is the coefficient related to the mechanism of drug release (Zamani et al., 2010; Nguyen et al., 2012; Gencturk et al., 2017). The dashed lines in Figure 5 show the fits of Equation 1 to the early time data below 60%. The “*a*” and “*n*” values and the error *R*^2^ obtained from the fits are given in Table 1.

**Table 1:** The “*a*” and “*n*” values obtained from the fit of Equation 1 to the release data of the coated and uncoated fibers.

	**Coated samples**			**Uncoated samples**		
	***R*^**2**^**	***n***	***a***	***R*^**2**^**	***n***	***a***
pH 4	0.9096	0.3288	0.3829	0.9986	0.7991	0.8151
pH 6.5	0.9044	0.4110	0.3870	0.9876	0.7589	0.8693
pH 9	0.9461	0.5206	0.5503	0.9979	0.7655	0.7758

The control experiments performed using the uncoated PVA-RB nanofibers reveal a fast-release kinetics at early times, which is not affected by the pH of the medium. The kinetic parameter “*n*” is found to be ~0.77, which indicates an anomalous transport mechanism, dominated by concentration-dependent diffusion and dissolution of the polymer. The release kinetics from the polymer-coated samples, on the other hand, reveals a pH dependent behavior. At all pH conditions, the values of “*n*” are less than the control samples, indicating that the Fickian diffusion dominates over the polymer dissolution mechanism in the coated samples (Fu and Kao, 2010). As the pH of the release medium increases, faster kinetics is observed as indicated by higher “*n*” values. Faster kinetics at high pH values can be attributed to the collapsed state of the coating, which leads to larger free volumes compared to low pH conditions.

Although Peppas model was used to fit the release data, it should be noted that the fit parameters obtained were mostly used to study the effect of the pH on the release kinetics and to comment on the dominant mechanisms, as opposed to thoroughly explaining the active mechanisms. Our system deviates from these models due to presence of an insoluble, pH-responsive polymer coating on the top and bottom layers of the mat. This coating impedes the full dissolution of the polymer nanofibers, introduces electrostatic interactions, and impacts the diffusion paths in the swollen state, thus affecting the release rate of the drug.

### Cellular Response to p(4VP-*co*-EGDMA)-Coated PVA-RB Nanofibers

#### Immunocytochemistry of Cells Exposed to the Coated PVA-RB Nanofibers

**Figure 7:**
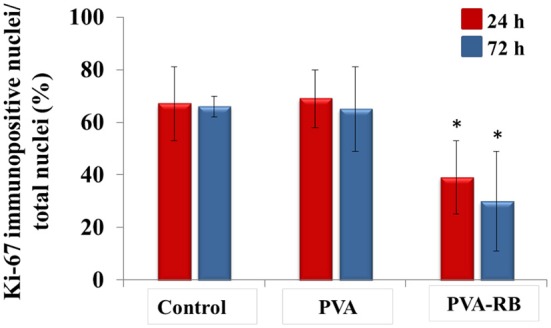
Immunocytochemical investigation of coated PVA and coated PVA-RB exposed cells by staining with Ki67 markers at 24 and 72 h of incubation (analyzed with Student *t*-test ^*^p < 0.05).

The proliferation ability of the cells exposed to the coated PVA and coated PVA-RB nanofibers were also elucidated by Ki67 antibody immunohistochemical staining that binds to the proliferating cell marker Ki67 antigens selectively. The results indicated that the decrease in the coated PVA nanofiber-exposed cells was insignificant while for the cells exposed to the coated PVA-RB nanofibers, cell proliferation decreased to 28% and 36% at the end of 24 and 72 h of incubations, respectively (Figure 7).

#### ROS Production in Cells Exposed to the Coated PVA-RB Nanofibers

**Figure 8:**
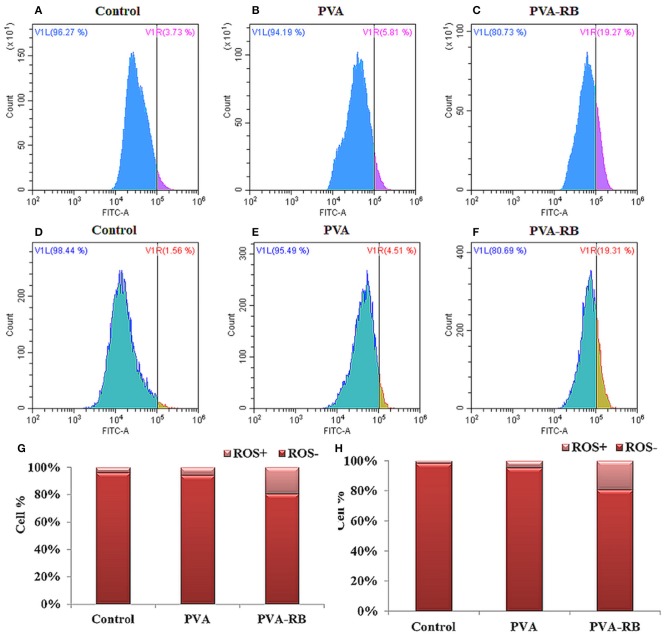
ROS production in control, coated PVA and coated PVA-RB nanofiber exposed U87MG cells at 24 h **(A–C)** and 72 h **(D–F)** of incubation. Percentage of ROS positive U87MG cells at 24 h **(G)** and 72 h **(H)** of incubation.

As a defense mechanism, cells have the tendency to increase ROS production when they are exposed to foreign substances. The increased ROS levels in mitochondria cause cellular stress, and then stimulate further ROS production. High ROS level in the cells activates apoptosis processes due to the hindered cellular functions by damaged critical cell components, such as proteins, membrane lipids, and DNA (Murphy, 2009). In the light of the natural defense mechanism of cells, chemotherapy drugs are generally designed as ROS stimulating agents to activate apoptosis process. In this study, coated PVA-RB nanofibers were investigated comparatively to coated PVA nanofibers on U87MG brain cancer cells following 24 and 72 h of incubation, as shown in Figure 8. The results indicated that significant ROS level increment in coated PVA-RB fiber-exposed cells was observed (19%) after 24 and 72 h of incubation, as shown in Figures 8C,E, respectively. However, the ROS level of coated PVA nanofiber-exposed cells was about 5% after 24 and 72 h of incubation, as shown in Figures 8B,D, respectively. High levels of ROS produced in the cells exposed to the coated PVA-RB nanofibers indicated that RB induce damage in cells, as expected from chemotherapy drugs.

#### Cell Death Mechanism of Cells Exposed to the Coated PVA-RB Nanofibers

**Figure 9:**
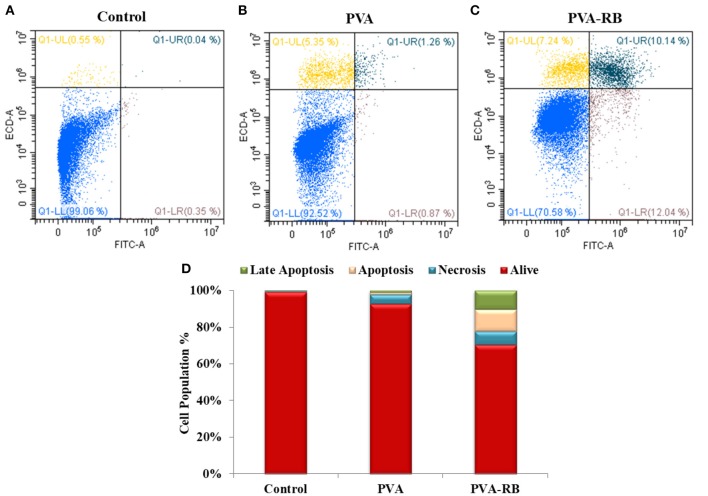
Cell death mechanism in control **(A)**, coated PVA **(B)** and coated PVA-RB **(C)** nanofiber exposed U87MG cells at 24 h of incubation. **(D)** Percentage of living, apoptotic and necrotic cells.

The optimal *in vitro* conditions for cell death that mimic the organism were provided by Fink and Cookson (2005) to analyze the effects of structures on the death mechanism. Effects of the coated PVA-RB and coated PVA nanofibers were investigated on U87MG brain cancer cells, which were labeled with annexin-V and PI as indicators of apoptosis and necrosis, as reported in Figure 9.

The results showed that of all the cells exposed to coated PVA-RB nanofibers, 70% was alive, while 10, 12, and 7% of cells were in late apoptosis, apoptosis, and necrosis after 24 h of incubation, respectively (Figure 9C). On the other hand, 92% of the cells exposed to coated PVA nanofiber-exposed cells were alive, while 5% of the cells were in necrosis and 1% of cells were in late apoptosis after 72 h of incubation (Figure 9B). The results confirmed that RB-loaded nanofibers cause serious apoptotic and late apoptotic effects in glioblastoma cells, as targeted in cancer therapy applications.

## Conclusion

A pH-responsive nanofiber mat loaded with RB was produced as a potential controlled drug delivery system for post-operational cancer treatments. A thin layer of pH-responsive cross-linked p(4VP-*co*-EGDMA) polymer was coated on PVA-RB blend nanofibers to attain pH response to the fibers and to tune the release kinetics. The vapor-based iCVD technique was successfully employed for the conformal deposition of the polymer coating without damaging the nanofiber mat. The coating layer enabled the control of the degradation and release kinetics of the nanofibers by tuning the pH of the medium. The iCVD was demonstrated to be a successful technique to coat the nanofibers with stimuli-responsive, functional polymer films to attain additional properties to the fibers.

The therapeutic efficiency of coated PVA-RB nanofibers against brain cancer was investigated comparatively against the coated PVA nanofibers, to analyze the anti-cancer effect of RB released from the fibers. The results indicated that coated PVA-RB nanofibers selectively decreased cell proliferation and stimulated cell death in apoptotic direction by increasing ROS production in the cells at long-term incubation as desired in chemotherapeutic applications.

## Data Availability Statement

All datasets generated for this study are included in the article/Supplementary Material.

## Author Contributions

SSa designed the experiments, prepared the samples, and wrote the manuscript. AT performed the iCVD coatings and contributed to data analysis and interpretation. EO and SSh contributed to the electrospinning process and performed the release studies. ME, GG, and OS performed the drug release studies of the nanofibers with *in vitro* cell culture experiments. GO and GC guided the study, wrote, and revised the manuscript.

### Conflict of Interest

The authors declare that the research was conducted in the absence of any commercial or financial relationships that could be construed as a potential conflict of interest. The reviewer CT-T declared a past collaboration with one of the authors GC to the handling editor.

## References

[B1] Aljehani A. K. Hussaini M. A. Hussain M. A. Alothmany N. S. Aldhaheri R. W. (2014). Effect of electrospinning parameters on nanofiber diameter made of poly (vinyl alcohol) as determined by Atomic Force Microscopy, in 2nd Middle East Conference on Biomedical Engineering (Doha: IEEE), 379–381. 10.1109/MECBME.2014.6783283

[B2] Armagan E. Ince G. (2015). Coaxial nanotubes of stimuli responsive polymers with tunable release kinetics. Soft Matter 11, 8069–8075. 10.1039/C5SM01074H 26333009

[B3] Bayari S. Yurdakul S. (2000). Fourier transform infrared and Raman spectra of 4-vinylpyridine and its transition metal (II) tetracyanonickelate complexes. Spectrosc. Lett. 33, 475–483. 10.1080/00387010009350132

[B4] Bazhban M. Nouri M. Mokhtari J. (2013). Electrospinning of cyclodextrin functionalized chitosan/PVA nanofibers as a drug delivery system. Chinese J. Polym. Sci. 31, 1343–1351. 10.1007/s10118-013-1309-5

[B5] Brown C. L. III. Crawford N. (2002). Iontophoresis Electroporation and Combination Patches for Local Drug Delivery to Body Tissues. U.S. Patent No. 6,424,862. Washington, DC: U.S. Patent and Trademark Office.

[B6] Chakraborty S. Liao I. C. Adler A. Leong K. W. (2009). Electrohydrodynamics: a facile technique to fabricate drug delivery systems. Adv. Drug Deliv. Rev. 61, 1043–1054. 10.1016/j.addr.2009.07.013 19651167 PMC2761154

[B7] Chunder A. Sarkar S. Yu Y. Zhai L. (2007). Fabrication of ultrathin polyelectrolyte fibers and their controlled release properties. Colloids Surf. B Biointerfaces 58, 172–179. 10.1016/j.colsurfb.2007.03.004 17418541

[B8] Croisier F. Atanasova G. Poumay Y. Jérôme C. (2014). Polysaccharide-coated PCL nanofibers for wound dressing applications. Adv. Healthc. Mater. 3, 2032–2039. 10.1002/adhm.201400380 25263074

[B9] Dabrzalska M. Benseny-Cases N. Barnadas-Rodríguez R. Mignani S. Zablocka M. Majoral J. P.. (2016). Fourier transform infrared spectroscopy (FTIR) characterization of the interaction of anti-cancer photosensitizers with dendrimers. Anal. Bioanal. Chem. 408, 535–544. 10.1007/s00216-015-9125-0 26507333

[B10] Fink S. L. Cookson B. T. (2005). Apoptosis, pyroptosis, and necrosis: mechanistic description of dead and dying eukaryotic cells. Infec. Immun. 73, 1907–1916. 10.1128/IAI.73.4.1907-1916.2005 15784530 PMC1087413

[B11] Fu Y. Kao W. J. (2010). Drug release kinetics and transport mechanisms of non-degradable and degradable polymeric delivery systems. Expert Opin. Drug Deliv. 7, 429–444. 10.1517/17425241003602259 20331353 PMC2846103

[B12] Garg T. Malik B. Rath G. Goyal A. K. (2014). Development and characterization of nano-fiber patch for the treatment of glaucoma. Eur. J. Pharm. Sci. 53, 10–16. 10.1016/j.ejps.2013.11.016 24333373

[B13] Gencturk A. Kahraman E. Güngör S. Özhan G. Özsoy Y. Sarac A. S. (2017). Polyurethane/hydroxypropyl cellulose electrospun nanofiber mats as potential transdermal drug delivery system: characterization studies and *in vitro* assays. Artif. Cells Nanomed. Biotechnol. 45, 655–664. 10.3109/21691401.2016.1173047 27103498

[B14] He C. L. Huang Z. M. Han X. J. Liu L. Zhang H. S. Chen L. S. (2006). Coaxial electrospun poly (L-lactic acid) ultrafine fibers for sustained drug delivery. J. Macromol. Sci. B 45, 515–524. 10.1080/00222340600769832

[B15] Huang R. Y. M. Rhim J. W. (1993). Modification of poly (vinyl alcohol) using maleic acid and its application to the separation of acetic acid-water mixtures by the pervaporation technique. Polym. Int. 30, 129–135. 10.1002/pi.4990300119

[B16] Jalvandi J. White M. Gao Y. Truong Y. B. Padhye R. Kyratzis I. L. (2017). Polyvinyl alcohol composite nanofibres containing conjugated levofloxacin-chitosan for controlled drug release. Mater. Sci. Eng. 73, 440–446. 10.1016/j.msec.2016.12.112 28183630

[B17] Jannesari M. Varshosaz J. Morshed M. Zamani M. (2011). Composite poly (vinyl alcohol)/poly (vinyl acetate) electrospun nanofibrous mats as a novel wound dressing matrix for controlled release of drugs. Int. J. Nanomed. 6, 993–1003. 10.2147/IJN.S17595 21720511 PMC3124403

[B18] Kenawy E. R. Abdel-Hay F. I. El-Newehy M. H. Wnek G. E. (2007). Controlled release of ketoprofen from electrospun poly (vinyl alcohol) nanofibers. Mater. Sci. Eng. 459, 390–396. 10.1016/j.msea.2007.01.039

[B19] Kenawy E. R. Bowlin G. L. Mansfield K. Layman J. Simpson D. G. Sanders E. H. (2002). Release of tetracycline hydrochloride from electrospun poly (ethylene-co-vinylacetate), poly (lactic acid), and a blend. J. Control. Release 81, 57–64. 10.1016/S0168-3659(02)00041-X 11992678

[B20] Koevary S. B. (2012). Selective toxicity of rose bengal to ovarian cancer cells *in vitro*. Int. J. Physiol. Pathophysiol. Pharmacol. 4, 99–107. 22837809 PMC3403562

[B21] Korsmeyer R. W. Gurny R. Doelker E. Buri P. Peppas N. A. (1983). Mechanisms of solute release from porous hydrophilic polymers. Int. J. Pharm. 15, 25–35. 10.1016/0378-5173(83)90064-9 6644570

[B22] LaPorte S. Haller M. Hooper W. Lent M. Riff K. Heruth K. (2005). Implantable Medical Device and Patch System and Method of Use. U.S. Patent Application No. 10/137,516.

[B23] Lau K. K. Gleason K. K. (2006). Initiated chemical vapor deposition (iCVD) of poly (alkyl acrylates): an experimental study. Macromolecules 39, 3688–3694. 10.1021/ma0601619

[B24] Lau K. K. Gleason K. K. (2007). All-dry synthesis and coating of methacrylic acid copolymers for controlled release. Macromol. Biosci. 7, 429–434. 10.1002/mabi.200700017 17429803

[B25] Li D. He Q. Yang Y. Möhwald H. Li J. (2008). Two-stage pH response of poly (4-vinylpyridine) grafted gold nanoparticles. Macromolecules 41, 7254–7256. 10.1021/ma800894c

[B26] Li W. Xu R. Zheng L. Du J. Zhu Y. Huang R.. (2012). LBL structured chitosan-layered silicate intercalated composites based fibrous mats for protein delivery. Carbohydr. Polym. 90, 1656–1663. 10.1016/j.carbpol.2012.07.046 22944430

[B27] Li X. Kanjwal M. A. Lin L. Chronakis I. S. (2013). Electrospun polyvinyl-alcohol nanofibers as oral fast-dissolving delivery system of caffeine and riboflavin. Colloids Surf. B Biointerfaces 103, 182–188. 10.1016/j.colsurfb.2012.10.016 23201736

[B28] Mansur H. S. Sadahira C. M. Souza A. N. Mansur A. A. (2008). FTIR spectroscopy characterization of poly (vinyl alcohol) hydrogel with different hydrolysis degree and chemically crosslinked with glutaraldehyde. Mater. Sci. Eng. 28, 539–548. 10.1016/j.msec.2007.10.088

[B29] Murphy M. P. (2009). How mitochondria produce reactive oxygen species. Biochem. J. 417, 1–13. 10.1042/BJ20081386 19061483 PMC2605959

[B30] Nelson K. D. Romero-Sanchez A. A. Smith G. M. Alikacem N. Radulescu D. Waggoner P. (2003). Drug Releasing Biodegradable Fiber Implant. U.S. Patent No. 6,596,296. Washington, DC: U.S. Patent and Trademark Office.

[B31] Nguyen T. T. T. Ghosh C. Hwang S. G. Chanunpanich N. Park J. S. (2012). Porous core/sheath composite nanofibers fabricated by coaxial electrospinning as a potential mat for drug release system. Int. J. Pharm. 439, 296–306. 10.1016/j.ijpharm.2012.09.019 22989981

[B32] Oliveira M. F. Suarez D. Rocha J. C. B. de Carvalho Teixeira A. V. N. Cortés M. E. De Sousa F. B.. (2015). Electrospun nanofibers of polyCD/PMAA polymers and their potential application as drug delivery system. Mater. Sci. Eng. 54, 252–261. 10.1016/j.msec.2015.04.042 26046289

[B33] Ozaydin-Ince G. Coclite A. M. Gleason K. K. (2011). CVD of polymeric thin films: applications in sensors, biotechnology, microelectronics/organic electronics, microfluidics, MEMS, composites and membranes. Rep. Prog. Phys. 75:016501. 10.1088/0034-4885/75/1/016501 22790306

[B34] Qin J. Kunda N. Qiao G. Calata J. F. Pardiwala K. Prabhakar B. S.. (2017). Colon cancer cell treatment with rose bengal generates a protective immune response via immunogenic cell death. Cell Death Dis. 8:e2584. 10.1038/cddis.2016.473 28151483 PMC5386459

[B35] Sakai S. Yamada Y. Yamaguchi T. Ciach T. Kawakami K. (2009). Surface immobilization of poly (ethyleneimine) and plasmid DNA on electrospun poly (L-lactic acid) fibrous mats using a layer-by-layer approach for gene delivery. J. Biomed. Mater. Res. A 88, 281–287. 10.1002/jbm.a.31870 18260146

[B36] Sharma A. Gupta A. Rath G. Goyal A. Mathur R. B. Dhakate S. R. (2013). Electrospun composite nanofiber-based transmucosal patch for anti-diabetic drug delivery. J. Mater. Chem. B 1, 3410–3418. 10.1039/c3tb20487a 32260931

[B37] Song W. Yu X. Markel D. C. Shi T. Ren W. (2013). Coaxial PCL/PVA electrospun nanofibers: osseointegration enhancer and controlled drug release device. Biofabrication 5:035006. 10.1088/1758-5082/5/3/035006 23799653

[B38] Taepaiboon P. Rungsardthong U. Supaphol P. (2006). Drug-loaded electrospun mats of poly (vinyl alcohol) fibres and their release characteristics of four model drugs. Nanotechnology 17, 2317–2329. 10.1088/0957-4484/17/9/041

[B39] Thakkar S. Misra M. (2017). Electrospun polymeric nanofibers: new horizons in drug delivery. Eur. J. Pharm. Sci. 107, 148–167. 10.1016/j.ejps.2017.07.001 28690099

[B40] Theeuwes F. Nelson T. S. (2004). Implantable Drug Delivery Patch. U.S. Patent No. 6,726,920. Washington, DC: U.S. Patent and Trademark Office.

[B41] Tipduangta P. Belton P. Fábián L. Wang L. Y. Tang H. Eddleston M.. (2015). Electrospun polymer blend nanofibers for tunable drug delivery: the role of transformative phase separation on controlling the release rate. Mol. Pharm. 13, 25–39. 10.1021/acs.molpharmaceut.5b00359 26655957

[B42] Toomey P. Kodumudi K. Weber A. Kuhn L. Moore E. Sarnaik A. A.. (2013). Intralesional injection of rose bengal induces a systemic tumor-specific immune response in murine models of melanoma and breast cancer. PLoS ONE 8:e68561. 10.1371/journal.pone.0068561 23874673 PMC3714270

[B43] Tserkovsky D. A. Alexandrova E. N. Chalau V. N. Istomin Yu P. (2012). Effects of combined sonodynamic and photodynamic therapies with photolon on a glioma c6 tumor model. Exp. Oncol. 34, 332–335. 23302991

[B44] Wang B. Wang Y. Yin T. Yu Q. (2010). Applications of electrospinning technique in drug delivery. Chem. Eng. Commun. 197, 1315–1338. 10.1080/00986441003625997

[B45] Wang Y. Kozlovskaya V. Arcibal I. G. Cropek D. M. Kharlampieva E. (2013). Highly swellable ultrathin poly (4-vinylpyridine) multilayer hydrogels with pH-triggered surface wettability. Soft Matter 9, 9420–9429. 10.1039/c3sm51496j

[B46] Xu X. Chen X. Xu X. Lu T. Wang X. Yang L.. (2006). BCNU-loaded PEG–PLLA ultrafine fibers and their *in vitro* antitumor activity against Glioma C6 cells. J. Control. Release 114, 307–316. 10.1016/j.jconrel.2006.05.031 16891029

[B47] Yang D. Li Y. Nie J. (2007). Preparation of gelatin/PVA nanofibers and their potential application in controlled release of drugs. Carbohydr. Polym. 69, 538–543. 10.1016/j.carbpol.2007.01.008

[B48] Zamani M. Morshed M. Varshosaz J. Jannesari M. (2010). Controlled release of metronidazole benzoate from poly ε-caprolactone electrospun nanofibers for periodontal diseases. Eur. J. Pharm. Biopharm. 75, 179–185. 10.1016/j.ejpb.2010.02.002 20144711

[B49] Zamani M. Prabhakaran M. P. Ramakrishna S. (2013). Advances in drug delivery via electrospun and electrosprayed nanomaterials. Int. J. Nanomedicine 8, 2997–3017. 10.2147/IJN.S43575 23976851 PMC3746732

[B50] Zeng J. Aigner A. Czubayko F. Kissel T. Wendorff J. H. Greiner A. (2005). Poly (vinyl alcohol) nanofibers by electrospinning as a protein delivery system and the retardation of enzyme release by additional polymer coatings. Biomacromolecules 6, 1484–1488. 10.1021/bm0492576 15877368

[B51] Zhang Y. Huang Z. M. Xu X. Lim C. T. Ramakrishna S. (2004). Preparation of core– shell structured PCL-r-gelatin bi-component nanofibers by coaxial electrospinning. Chem. Mater. 16, 3406–3409. 10.1021/cm049580f

[B52] Zupancic S. Sinha-Ray S. Sinha-Ray S. Kristl J. Yarin A. L. (2016). Controlled release of ciprofloxacin from core–shell nanofibers with monolithic or blended core. Mol. Pharm. 13, 1393–1404. 10.1021/acs.molpharmaceut.6b00039 26950163

