# Supplementary material for: Electrospun Nanofibers With pH-Responsive Coatings for Control of Release Kinetics
Source: Front Bioeng Biotechnol. 2019 Nov 27;7:309. doi: 10.3389/fbioe.2019.00309 (PMC6892405; doi:10.3389/fbioe.2019.00309)
Supplement: Supplementary file 1 [file Data_Sheet_1.docx]

**SUPPORTING INFORMATION**

The mesh size determines the diffusion of Rose Bengal molecules through the coating. The average molecular weight,$\bar{M}_{c}$, which is related to the mesh size, can be calculated using [1]

$$\frac{1}{\bar{M_{c}}}=\frac{2}{\bar{M_{n}}}-\frac{\frac{\bar{\nu}}{V}\left[ Ln\left( 1-\nu_{s} \right)+\nu_{s}+\chi\nu_{s}^{2} \right]{[1-\frac{M_{r}}{2\bar{M}_{c}}\nu_{s}^{\frac{2}{3}}]}^{3}}{[\nu_{s}^{\frac{1}{3}}-\frac{1}{2}\nu_{s}]{[1+\frac{M_{r}}{2\bar{M}_{c}}\nu_{s}^{\frac{1}{3}}]}^{2}}$$

where $\bar{M}_{n}$ is the number average molecular weight which is large enough so that the first term on the right hand side can be neglected [2]. V is the molar volume of water (18 cm^3^/mol), $M_{r}$ is the molecular weight of the 4-vinyl pyridine repeat unit (105.14 g/mol) and $\bar{\nu}$is the specific volume of p(4-vinyl pyridine) (1.012 cm^3^/g), χ is the Flory-Huggins interaction parameter and is taken as 0.6 . The ratios of the dry to wet thickness of the polymers, denoted as$\nu_{s}$, are obtained from ellipsometry measurements performed on polymer films deposited on Si substrates and they are 0.617, 0.952 and 0.952 at pH 4, pH 6.5 and pH 9, respectively.

The mesh size, ξ, of the deposited polymers can be determined using the relation [3, 4]

$$\xi=\nu_{s}^{\frac{-1}{3}}l{(\frac{2\bar{M}_{c}}{M_{r}})}^{\frac{1}{2}}C_{n}^{\frac{1}{2}}$$

where $C_{n}$is the characteristic ratio for 4-vinyl pyridine, which is taken as 10 [5] and l is the C-C bond length, which for vinyl polymers is 0.154 nm [6]. The mesh sizes of the coating at pH 4, pH 6.5 and pH 9 are calculated as 1.16 nm, 0.69 nm and 0.69 nm, respectively.

Rose Bengal hydrodynamic radius is independent of pH values and it is 1.28nm [7].

1. Peppas, N.A. and E.W. Merrill, *Crosslinked poly (vinyl alcohol) hydrogels as swollen elastic networks.* Journal of Applied Polymer Science, 1977. **21**(7): p. 1763-1770.

2. Baxamusa, S.H., et al., *Protection of sensors for biological applications by photoinitiated chemical vapor deposition of hydrogel thin films.* Biomacromolecules, 2008. **9**(10): p. 2857-2862.

3. Peppas, N.A., et al., *Hydrogels in biology and medicine: from molecular principles to bionanotechnology.* Advanced materials, 2006. **18**(11): p. 1345-1360.

4. Billmeyer, F.W. and F.W. Billmeyer, *Textbook of polymer science.* 1984.

5. Yang, S., M. Hassan, and P. Akcora, *Role of adsorbed chain rigidity in reinforcement of polymer nanocomposites.* Journal of Polymer Science Part B: Polymer Physics, 2019. **57**(1): p. 9-14.

6. Koetting, M.C., et al., *Stimulus-responsive hydrogels: Theory, modern advances, and applications.* Materials Science and Engineering: R: Reports, 2015. **93**: p. 1-49.

7. Vlasova, I., et al., *Investigation of the rotational diffusion of the Rose Bengal fluorescent nanomarker in human serum albumin solutions.* Moscow University Physics Bulletin, 2013. **68**(3): p. 231-234.
